# Disparities in Genetic Diversity Drive the Population Displacement of Two Invasive Cryptic Species of the *Bemisia tabaci* Complex in China

**DOI:** 10.3390/ijms25147966

**Published:** 2024-07-21

**Authors:** Yantao Xue, Yusheng Wang, Jiqiang Chen, Guifen Zhang, Wanxue Liu, Fanghao Wan, Yibo Zhang

**Affiliations:** 1 State Key Laboratory for Biology of Plant Diseases and Insect Pests, Institute of Plant Protection, Chinese Academy of Agricultural Sciences, Beijing 100193, China; yantao_xue@163.com (Y.X.); wangyusheng@hunau.edu.cn (Y.W.); chenjiqiang1126@163.com (J.C.); zhangguifen@caas.cn (G.Z.); liuwanxue@caas.cn (W.L.); wanfanghao@caas.cn (F.W.); 2 College of Plant Protection, Hunan Agricultural University, Changsha 410128, China

**Keywords:** invasive alien pests, genetic diversity, population structure, mitochondrial DNA, microsatellites

## Abstract

Within the whitefly *Bemisia tabaci* (Gennadius) (Hemiptera: Aleyrodidae) complex, two cryptic species, namely Middle East-Asia Minor 1 (MEAM1) and Mediterranean (MED), are important invasive pests affecting global agriculture and horticulture. They were introduced into China sequentially in the mid-1990s and around 2003, respectively. Subsequently, the latter invader MED has outcompeted the earlier invader MEAM1, becoming the dominant population in the field. Although extensive studies have explored the underlying mechanisms driving this shift, the contribution of population genetics remains notably underexplored. In this study, we analyzed the genetic diversity and structure of 22 MED and 8 MEAM1 populations from various regions of China using mitochondrial DNA sequencing and microsatellite genotyping. Our results indicate low and moderate levels of genetic differentiation among geographically separate populations of MED and MEAM1, respectively. Median-joining network analysis of *mtCOI* gene haplotypes revealed no clear geographic structuring for either, with common haplotypes observed across provinces, although MED had more haplotypes. Comparative analyses revealed that MED presented greater genetic diversity than MEAM1 on the basis of two markers. Furthermore, analysis of molecular variance supported these findings, suggesting that while some genetic variation exists between populations, a significant amount is also present within populations. These findings reveal the population genetics of the two invasive cryptic species of the *B. tabaci* complex in China and suggest that the disparities in genetic diversity drive the displacement of their populations in the field. This work also provides valuable information on the genetic factors influencing the population dynamics and dominance of these invasive whitefly species.

## 1. Introduction

Invasive species are a leading cause of biodiversity loss and ecosystem disruption worldwide, necessitating an in-depth understanding of the mechanisms underlying successful invasions [1]. The dynamics of species dominance within ecological niches are complex, with population genetics playing a crucial role in determining the outcome of species interactions [2]. For nonnative species, genetic diversity is often a key determinant of their invasiveness and potential to spread and establish in novel habitats [3,4]. High levels of genetic variation can facilitate rapid adaptation to novel environments, predisposing some populations to becoming invasive [5]. Therefore, understanding the genetic underpinnings of successful invasion and competitive displacement of populations is critical to advancing our understanding of invasion mechanisms.

The whitefly *Bemisia tabaci* (Gennadius) (Hemiptera: Aleyrodidae), also known as the silverleaf whitefly or sweet potato whitefly, provides an interesting case study for dissecting the genetic factors that contribute to invasive success and population displacement. It is one of the most widespread and insidious pests plaguing agriculture and horticulture worldwide [6] and is notorious for its ability to transmit plant viruses, cause direct damage through sap feeding, and induce the growth of sooty mold on plants, causing severe economic losses globally [7,8]. This pest is generally considered to consist of a complex of more than 44 cryptic species that are morphologically identical but exhibit significant variation in biological traits, such as the ecological niche, plant host range, endosymbionts, insecticide resistance, and ability to transmit viruses [9,10,11,12,13,14].

The invasive nature of two members within this complex, namely, Middle East-Asia Minor 1 (MEAM1, formerly known as the B biotype) and Mediterranean (MED, formerly known as the Q biotype), has led to widespread concern among both researchers and agricultural practitioners [15,16,17]. In China, these two invasive whiteflies were successively discovered in the mid-1990s and early 21st century (approximately 2003) [18,19]. Following a succession of events in which MEAM1 replaced native cryptic species populations and was subsequently displaced by MED populations, the latter invader MED became the dominant cryptic species in most Chinese field regions by 2020 [20]. Despite extensive research aimed at elucidating the ecological and biological mechanisms contributing to this shift, such as insecticide application, host plants, feeding behavior, and environmental suitability [21,22,23], the specific role of population genetics in driving the dynamics of invasion and dominance between these two species has not been fully explored.

Exploring the molecular variability within species is a useful approach for population genetic studies [24]. Currently, molecular markers such as mitochondrial DNA and microsatellites are widely used for species identification and population genetics studies [25]. Owing to their high resolution in revealing genetic diversity, mitochondrial DNA markers, specifically the cytochrome *c* oxidase subunit I (*mtCOI*) gene, have been widely applied in DNA barcoding [26,27]. Microsatellites, another type of molecular marker, exhibit high polymorphism and codominance, making them valuable for evaluating genetic diversity, population structure, and gene flow patterns both within and among populations [28,29]. By integrating both types of markers, a comprehensive analysis of intra- and interspecific variation can be performed [30,31,32].

In this study, we used mitochondrial DNA sequencing and microsatellite genotyping techniques to analyze the genetic diversity and population structure of 22 MED and 8 MEAM1 populations collected from different regions of China. We not only investigated the levels of genetic differentiation and gene flow between and among populations of both species, shedding light on the geographical structuring of genetic variation but also compared their genetic diversities. On this basis, we elucidated whether the successful invasion and competitive displacement of *B. tabaci* MED and MEAM1 in China has been facilitated by disparities in their population genetics. This work provides crucial information for developing informed strategies to manage these destructive pests and to advance our understanding of genetic influences on species invasiveness and competitiveness more broadly.

## 2. Results

### 2.1. Genetic Diversity

**Figure 1:**
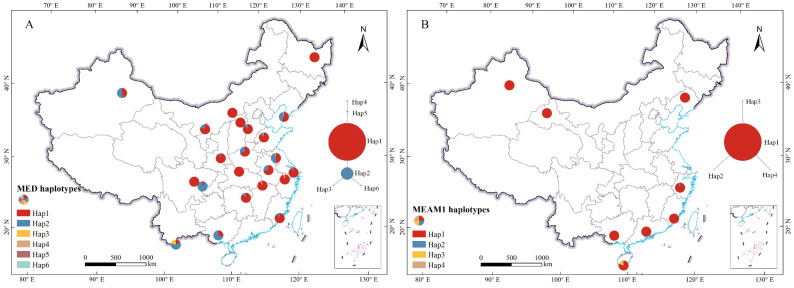
Sampling locations, haplotype frequencies, and median-joining haplotype networks based on the *mtCOI* gene sequences of *Bemisia tabaci *MED (**A**) and MEAM1 (**B**) in China.

In total, 405 MED individuals and 127 MEAM1 individuals were obtained from 22 and 8 sites, respectively (Table 1, Figure 1). A 756 bp fragment of the *mtCOI* gene was obtained from the MED and MEAM1 populations individually. In the MED population sequences, there were 751 constant sites and 5 variable sites, of which 4 were parsimony-informative sites and 1 was a singleton site. The *h* values ranged from 1 to 3, with average *Hd*, *π*, and *k* values of 0.401, 0.00058, and 0.441, respectively (Table 2). For MEAM1, there were 753 constant sites and 3 variable sites, of which 1 was a parsimony-informative site and 2 were singleton sites, with *h* values ranging from 1 to 3 and average *Hd*, *π*, and *k* values of 0.064, 0.00009, and 0.065, respectively (Table 2). Greater genetic diversity was observed in the Jiangsu (JS) population of MED (*Hd* = 0.567, *π* = 0.00081, and *k* = 0.615) and the Hainan (HI) population of MEAM1 (*Hd* = 0.362, *π* = 0.0005, and *k* = 0.381).

**Figure 2:**
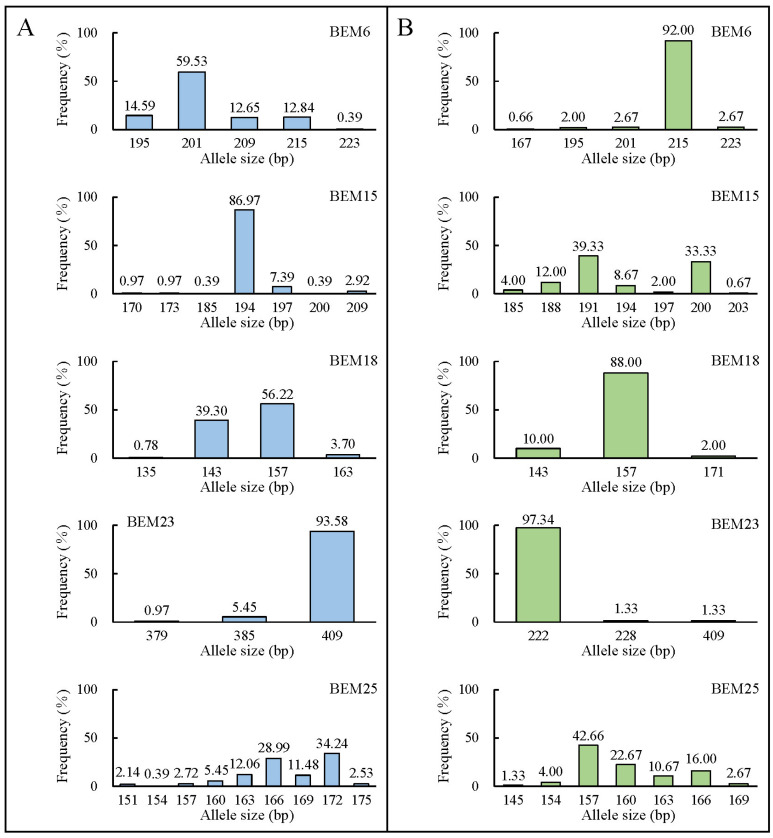
The allele size and frequency of five microsatellite loci in *B. tabaci* MED (**A**) and MEAM1 (**B**).

Microsatellite genotyping data from five microsatellite loci revealed differences in allele size and frequency between the two cryptic species at all loci (Figure 2). Across 22 MED populations, the *A* values per locus ranged from 3 to 9, with *Homs* ranging from 125 to 249 and *Hets* ranging from 8 to 132 (Table 3). For the 8 MEAM1 populations, the *A* values ranged from 3 to 7, with *Homs* ranging from 19 to 72 and *Hets* ranging from 3 to 56 (Table 3). The BEM25 locus had the highest *A*_R_ values of 4.863 for MED and 4.167 for MEAM1, whereas the BEM23 locus had the lowest *A*_R_ values of 1.605 for MED and 1.259 for MEAM1 (Table 3). In parallel, there was significant genetic variation within their respective populations (Table 2).

### 2.2. Haplotype Network

A total of six haplotypes were identified for MED, and four were identified for MEAM1 across all their *mtCOI* sequences. For MED, Hap1 was the predominant haplotype, represented by 73.7% of individuals and present in all populations. Four other haplotypes (Hap2, Hap3, Hap4, and Hap5) were found in more than one population, whereas Hap6 was unique to a single population (Figure 1A, Table 4). In MEAM1, Hap1 was the most common haplotype, represented by 96.75% of the individuals, whereas the remaining three haplotypes (Hap2, Hap3, and Hap4) were found in only one population each (Figure 1B, Table 5). The median-joining networks revealed a star-shaped radiation distribution for the MED and MEAM1 haplotypes, centered on the dominant haplotype, with other haplotypes differing by only one or two mutations (Figure 1). No clear geographic clustering of their haplotypes was observed, as both MED and MEAM1 presented random distributions of *mtCOI* haplotypes (Figure 1, Table 2).

### 2.3. Genetic Differentiation

**Figure 3:**
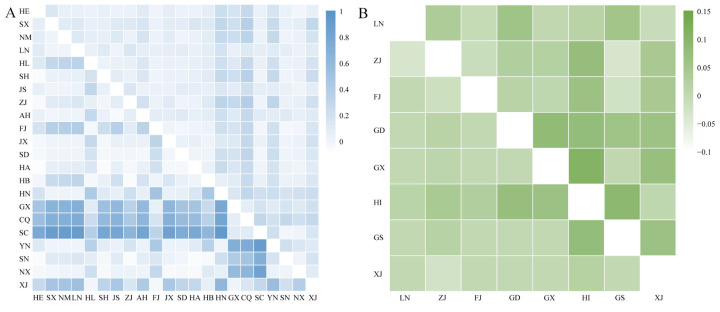
The pairwise fixation index (*F*_ST_) among different populations of *B. tabaci* MED (**A**) and MEAM1 (**B**) in China on the basis of *mtCOI* gene sequences (below the diagonal) and five microsatellite loci (above the diagonal).

The pairwise *F_ST_* values indicated moderate genetic differentiation among the MED populations (*mtCOI* differentiation: −0.024 to 0.384, mean = 0.106; microsatellite differentiation: −0.072 to 0.927, mean = 0.224) and weak differentiation among the MEAM1 populations (*mtCOI* data: −0.032 to 0.107, mean = 0.036; microsatellite data: −0.032 to 0.078, mean = 0.010) (Figure 3). Analysis of molecular variance (AMOVA) revealed that most genetic variance occurred within populations for both MED (*mtCOI* variation: 60.5%; microsatellite variation: 88.2%) and MEAM1 (*mtCOI* variation: 96.3%; microsatellite variation: 95.4%), with less variance occurring between populations (Table 6). Significant differences were observed between specific MED populations, such as Guangxi (GX), Chongqing (CQ), and Sichuan (SC), and most others. Neutrality tests via *Tajima’s* D and *Fu’s* F statistics revealed significantly negative values for both MED and MEAM1 (Table 1), suggesting demographic expansion in these populations.

### 2.4. Population Genetic Structure

**Figure 4:**
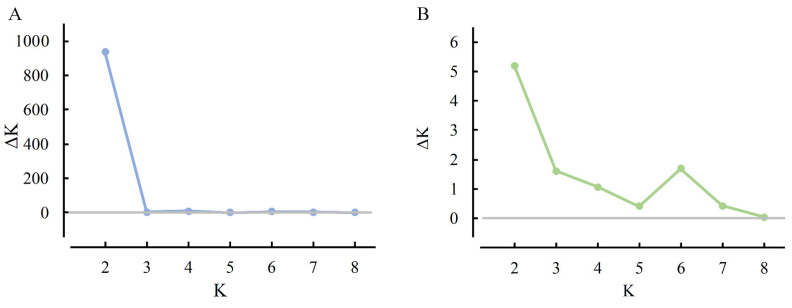
Line graph of the number of genetic clusters (K) by ΔK for different populations of *B. tabaci* MED (**A**) and MEAM1 (**B**) in China.

**Figure 5:**
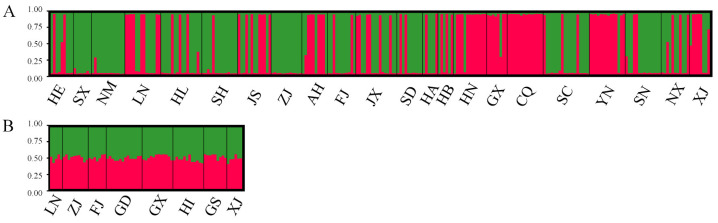
Genetic structure of the populations of *B. tabaci* MED (**A**) and MEAM1 (**B**) in China.

Structural analysis of the microsatellite data suggested that the optimal number of genetic clusters (K) was two for both cryptic species (Figure 4). This result implies that the 22 MED and 8 MEAM1 populations could be divided into two distinct genetic groups. In MED, specific populations, such as Shanxi (SX), Inner Mongolia (NM), and Zhejiang (ZJ), were dominated by one genetic group, whereas the Henan (HN), Guangxi (GX), and Yunnan (YN) populations had more from the other group, with other populations having individuals from both groups (Figure 5A). In MEAM1, however, each individual across all populations had nearly equal proportions of the two genetic groups (Figure 5B), indicating that MED populations in China are structured into two major clades, whereas the MEAM1 populations lack a strong phylogeographic structure and exist as a single clade in China.

## 3. Discussion

*B. tabaci* has increasingly been shown to exhibit complex genetic diversity, mostly as a unique cryptic species complex [33]. However, few studies have focused on the population genetics of the two invasive cryptic species within this complex, and even less is known about the role of genetic factors in their invasion and population displacement [34]. In this study, our genetic analysis revealed that the MED population presented greater genetic diversity than did the MEAM1 population in China, which is consistent with their population dominance and substitution process. Therefore, we hypothesize that disparities in genetic diversity are likely to have driven population shifts between these two invasive whiteflies.

The results of our analysis on the basis of the *mtCOI* gene indicate that both the MED and MEAM1 populations present limited genetic variation at this mitochondrial locus, despite their wide geographic distribution. This low genetic diversity could be due to various factors, such as recent population expansions following bottleneck events, selective sweeps, or a combination thereof [31,35]. The significant negative values obtained from *Tajima’s* D and *Fu’s* F statistics are consistent with a demographic expansion scenario. Nuclear genetic diversity, however, as indicated by microsatellite data, is more substantial within these populations. The discrepancy between mitochondrial and nuclear genetic diversity may stem from the different modes of inheritance and evolutionary dynamics between these two types of markers. Mitochondrial DNA is inherited exclusively through the maternal line, whereas microsatellites reflect biparental inheritance and may be assessed to capture a more complete history of genetic exchange within and among populations [36].

Previous studies on the basis of the *mtCOI* gene have shown that the MED population in China has low haplotype diversity and does not form significant geographic clusters, although global populations can be distinguished into two subclades [32,37,38]. The present study also confirmed those previous observations. The star-shaped radiation distribution in the haplotype network implies a common ancestor for the haplotypes, with subsequent mutations leading to the current diversity. The dominance of a single haplotype in MED populations may suggest a founder effect, where a small number of individuals colonized new areas and gave rise to the current populations. Nevertheless, the microsatellite marker analysis results in this study suggested that there may be two subdivisions of MED populations in China, while the population structure was not distinguished by geographic barriers. Similarly, Simón et al. [39] reported substantial population structure differences among MED populations on the basis of microsatellite marker analysis. In contrast, there may be a more homogenous population with less geographic influence on the genetic structure of MEAM1. Such homogeneity among different populations could result from extensive gene flow. Indeed, pairwise fixation index values revealed that there was no significant correlation between the evolutionary relationships of the haplotypes and the distributions of geographic populations of both MED and MEAM1. In addition, the results of AMOVA and structural analysis also provided no evidence for the clustering of geographic population structure. The low genetic diversity observed in invasive species could be influenced by genetic bottlenecks, founder effects, and insecticide use [40,41,42]. The persistence of a single population may also be due to its dispersal following annual population explosions [35], as our results show that the northern populations of MEAM1 have only one haplotype.

The present study, which was based on analysis of *mtCOI* gene sequences and microsatellite markers, revealed that the genetic diversity of MED was greater than that of MEAM1, which is consistent with the results of other reports based on analysis of AFLP or RAPD markers in China [43,44] as well as in the United States [29]. Although the results derived from these different molecular tools were not perfectly consistent regarding genetic diversity and gene flow between different geographic populations, the results consistently confirmed the objective disparities in genetic diversity between the two invasive cryptic species. This difference has been suggested to affect the invasiveness of these whitefly populations [43]. As corroborated by a field survey, MED has replaced MEAM1 as the dominant whitefly throughout China [20], as MED was the most prevalent cryptic species among the sampled sites in that study.

Interestingly, there are important biological differences between the two cryptic species. MEAM1 outperforms MED in terms of fecundity, nymphal survival, and population increase, aided by reproductive disturbance [45]. Thus, MEAM1 tended to outcompete and displace MED in the absence of anthropogenic disturbance. However, the relatively high insecticide resistance of MED gives it a competitive advantage over MEAM1 in the field [13,42]. As discussed above, insecticide use can significantly influence the genetic diversity of *B. tabaci* populations, potentially explaining the lower genetic variation observed. Nevertheless, MED exhibits relatively high genetic diversity, suggesting a greater capacity to adapt to changes in the external environment, including exposure to pesticides. Therefore, under the selective pressure of extensive and frequent insecticide applications, MED can prevail in competition with MEAM1, leading to significant shifts in dominance in field populations.

While previous research has revealed the global dispersal capability of MED and MEAM1 populations, this study presents a new distribution map and directly compares the genetic diversity of these two invasive species across China, providing new perspectives on the genetic basis of their invasion success and competitive displacement. These results highlight the importance of considering genetic diversity when formulating management strategies, as it may influence the adaptability and dispersal potential of populations. Further research should explore the role of host plants and environmental factors in shaping the genetic structure of these populations to provide insights into the ecology and evolution of these invasive species. Because the biotypes and genetic structure of *B. tabaci* can change rapidly over time, future research should also include long-term monitoring to track changes in population genetic structure.

## 4. Materials and Methods

### 4.1. Sample Collection and Identification

Adult *B. tabaci* whiteflies were collected from different provinces in China during the main growing season. In each province, two representative sites known for *B. tabaci* presence were selected, and at least 20 adult whiteflies were randomly collected from different host plants at each site. The samples were stored in 95% ethanol at −20 °C until DNA extraction. Genomic DNA was extracted individually according to the protocol described by De Barro et al. [6]. Cryptic species identification was further determined through DNA barcoding by sequencing a fragment of the *mtCOI* gene (see details below). If both sites within a province contained the same cryptic species, individuals from either site were selected for subsequent analysis.

### 4.2. Mitochondrial DNA Amplification and Sequencing

The *mtCOI* gene fragment of *B. tabaci* was amplified via PCR via a primer pair consisting of a forward primer (5′-TGRTTYTTTGGTCATCCVGAAGT-3′) and a reverse primer (5′-TTACTGCACTTTCTGCCACATTAG-3′). Each PCR mixture was prepared to a final volume of 25 μL, containing 2 μL of 10×Easy *Taq* buffer (+Mg^2+^), 2.5 μL of dNTP mixture (2.5 μM each), 1 μL of each primer (10 μM), 0.2 μL of *Taq* DNA Polymerase (2.5 U/μL), 2.0 μL of DNA template, and 16.3 μL of distilled water. The amplification conditions were as follows: initial denaturation at 94 °C for 4 min; 30 cycles of denaturation at 94 °C for 30 s, annealing at 55 °C for 45 s, and extension at 72 °C for 2 min; and a final extension at 72 °C for 7 min. The amplified products were visualized via electrophoresis on a 2% agarose gel, purified, and then sequenced in both directions by Sangon Biotech Co., Ltd. (Shanghai, China) via the Sanger sequencing method.

### 4.3. Microsatellite Amplification and Genotyping

The microsatellite loci BEM6, BEM15, BEM18, BEM23, and BEM25 identified by De Barro et al. [46] were validated according to their proposed methods and criteria. Each locus’s forward primer was labeled with the fluorescent dye FAM at the 5’ end. For these isolated microsatellites, the genotyping PCR was performed with a 50 μL reaction volume consisting of 25 μL of 2×T5 PCR Super PCR Mix for PAGE, 2 μL of reverse primer (10 μM), 2 μL of forward primer (10 μM), 1 μL of DNA template, and 20 μL of distilled water. The thermal cycling began with an initial denaturation step at 98 °C for 3 min, followed by 30 cycles of 98 °C for 10 s, 50 °C or 55 °C for 10 s, and 72 °C for 15 s, and a final extension at 72 °C for 2 min. After amplification, the products were visualized at Tsingke Biotechnology Co., Ltd. (Beijing, China). Allele profiles were identified via GeneMapper version 4.0 (Applied Biosystems, Foster, CA, USA).

### 4.4. Mitochondrial Data Analyses

The sequences of the *mtCOI* gene fragments were aligned using CLUSTAL W [47] implemented in MEGA X with the default multiple alignment parameters, and the alignments were then manually corrected by examining the chromatogram peaks to ensure accuracy [48]. Nucleotide composition and sequence variation information were analyzed via MEGA X software [48]. Population genetic diversity indices, including the number of haplotypes (*h*), haplotype diversity (*Hd*), nucleotide diversity (*π*), and average number of nucleotide differences (*k*), were calculated in DnaSP 6 [49]. Median-joining networks of *mtCOI* haplotypes were constructed via NETWORK 5.0.0.3 [50]. Fixation index (*F*_ST_) values between populations were estimated via Arlequin 3.5 [51]. AMOVA was performed to assess the genetic structure among different populations using Arlequin 3.5, yielding the sum of squares, variance components, and percentage of variation in the genetic variation within and between populations [51]. Two neutrality tests, *Tajima’s* D and *Fu’s* F, were applied across all populations to analyze changes in demographic history using DnaSP 6 [49].

### 4.5. Microsatellite Data Analyses

Genotype data were corrected using Micro-Checker 2.2.3 [52]. Genetic diversity per locus was evaluated by counting the number of alleles (*A*), the number of homozygotes (*Homs*), and the number of heterozygotes (*Hets*) using GenePop 3.2 [53]. Allelic richness (*A*_R_) and null allele frequencies were estimated using FSTAT 2.9.3 and the FreeNA program, respectively [54]. The size and frequency of all loci that were allelic in the MED and MEAM1 populations were examined. Population genetic diversity indices, including the observed number of alleles (*N*_a_), effective number of alleles (*N*_e_), Shannon’s information index (*I*), observed heterozygosity (*H*_o_), and expected heterozygosity (*H*_e_), were determined using POPGENE 1.32 [55]. The inbreeding coefficient (*F*_IS_) across all loci was calculated with FSTAT 2.9.3 [54]. Departures from the Hardy-Weinberg equilibrium (HWE) were assessed via exact tests in GenePop 3.2 [53]. AMOVA and *F*_ST_ calculations were carried out with Arlequin 3.5 [38]. For each cryptic species, the population genetic structure was estimated via the Bayesian model-based clustering method in STRUCTURE 2.3.4 [56]. The admixture model for individual ancestry was employed to assign hybrid individuals to population clusters. Ten independent runs were executed for K values ranging from 1 to 8, with a burn-in of 100,000 iterations followed by 500,000 Markov chain Monte Carlo (MCMC) replicates. The optimal number of groups (K) was determined via the change in the likelihood function (ΔK), which was calculated by submitting the results to Structure Harvester (http://taylor0.biology.ucla.edu/structureHarvester (accessed on 23 July 2022)). CLUMPP 1.1.2b software was used for model averaging of individual ancestry coefficients across the ten independent runs [57]. Finally, the clusters were visualized via DISTRUCT 1.1 [58].

## Figures and Tables

**Table 1:** Sampling information for *B. tabaci MED* and MEAM1 in China.

Cryptic Species	Population Code	Collection Location	Coordinates
MED	HE	Hebei, Shijiazhuang	38°10′ N, 114°30′ E
	SX	Shanxi, Xinzhou	39°11′ N, 113°15′ E
	NM	Inner Mongolia, Hohhot	40°42′ N, 111°50′ E
	LN	Liaoning, Dalian	39°14′ N, 121°43′ E
	HL	Heilongjiang, Mudanjiang	46°48′ N, 130°24′ E
	SH	Shanghai, Pudong	30°59′ N, 121°46′ E
	JS	Jiangsu, Huaian	33°30′ N, 119°06′ E
	ZJ	Zhejiang, Hangzhou	30°12′ N, 120°05′ E
	AH	Anhui, Hefei	31°53′ N, 117°28′ E
	FJ	Fujian, Xiamen	24°39′ N, 118°18′ E
	JX	Jiangxi, Jiujiang	29°44′ N, 116°7′ E
	SD	Shandong, Jinan	36°41′ N, 117°23′ E
	HA	Henan, Zhengzhou	34°55′ N, 113°36′ E
	HB	Hubei, Xiangyang	32°05′ N, 112°17′ E
	HN	Hunan, Changsha	28°10′ N, 113°07′ E
	GX	Guangxi, Nanning	23°43′ N, 106°48′ E
	CQ	Chongqing city, Tongnan	30°04′ N, 105°49′ E
	SC	Sichuan, Chengdu	30°49′ N, 104°21′ E
	YN	Yunnan, Xishuangbanna	21°27′ N, 101°34′ E
	SN	Shaanxi, Xi’an	34°10′ N, 109°08′ E
	NX	Ningxia, Yinchuan	38°28′ N, 106°22′ E
	XJ	Xinjiang, Urumqi	43°49′ N, 87°34′ E
MEAM1	LN	Liaoning, Shenyang	41°51′ N, 123°25′ E
	ZJ	Zhejiang, Jinhua	29°05′ N, 119°39′ E
	FJ	Fujian, Xiamen	24°39′ N, 118°18′ E
	GD	Guangdong, Guangzhou	23°09′ N, 113°23′ E
	GX	Guangxi, Nanning	23°43′ N, 106°48′ E
	HI	Hainan, Sanya	18°18′ N, 109°32′ E
	GS	Gansu, Jiuquan	40°31′ N, 95°47′ E
	XJ	Xinjiang, Urumqi	43°49′ N, 87°34′ E

**Table 2:** Genetic diversity of different populations of *B. tabaci* MED and MEAM1 in China.

Cryptic Species	Population	Mitochondrial *COI*							Microsatellites						
		*h*	*Hd*	*π*	*k*	*Tajima’s* D	*Fu’s* F		*Na*	*Ne*	*I*	*Ho*	*He*	*FIS*	*p* Value (HWE)
MED	HE	2	0.264	0.00035	0.264	−0.341	0.186		3.0	1.673	0.620	0.311	0.342	0.148	0.281
	SX	1	—	—	—	—	—		2.6	1.993	0.608	0.314	0.341	0.154	0.400
	NM	1	—	—	—	—	—		3.0	2.038	0.748	0.339	0.415	0.224	0.020
	LN	3	0.547	0.00078	0.593	0.261	0.280		3.0	2.282	0.762	0.314	0.417	0.281	0.000
	HL	1	—	—	—	—	—		3.4	1.755	0.695	0.288	0.386	0.284	0.008
	SH	2	0.138	0.00018	0.138	−0.741	−0.380		3.0	1.751	0.570	0.343	0.301	−0.104	0.417
	JS	3	0.567	0.00081	0.615	0.273	0.256		3.0	2.113	0.720	0.292	0.396	0.300	0.000
	ZJ	2	0.133	0.00035	0.267	−1.491	0.235		2.8	1.635	0.518	0.267	0.284	0.104	0.714
	AH	3	0.473	0.00067	0.505	−0.532	−0.465		2.8	2.114	0.679	0.280	0.379	0.310	0.002
	FJ	2	0.209	0.00028	0.209	−0.529	−0.011		3.0	2.102	0.685	0.309	0.369	0.207	0.058
	JX	3	0.177	0.00035	0.264	−1.175	−1.310		2.8	2.001	0.655	0.325	0.373	0.159	0.001
	SD	3	0.267	0.00049	0.371	−1.457	−0.728		3.0	1.858	0.672	0.360	0.375	0.092	0.429
	HA	2	0.467	0.00062	0.467	0.820	0.818		2.4	1.791	0.620	0.300	0.372	0.280	0.062
	HB	1	—	—	—	—	—		2.6	2.182	0.725	0.367	0.433	0.241	0.322
	HN	2	0.248	0.00033	0.248	−0.399	0.133		2.8	1.996	0.655	0.308	0.363	0.191	0.135
	GX	2	0.425	0.00056	0.425	0.870	1.039		4.0	2.157	0.924	0.450	0.484	0.137	0.141
	CQ	2	0.148	0.0002	0.148	−0.714	−0.317		3.4	2.012	0.710	0.343	0.365	0.098	0.268
	SC	1	—	—	—	—	—		4.0	2.278	0.804	0.289	0.402	0.307	0.000
	YN	3	0.560	0.00083	0.626	−0.011	−0.072		3.4	2.139	0.812	0.371	0.441	0.193	0.019
	SN	2	0.133	0.00018	0.133	−1.159	−0.649		3.0	2.138	0.742	0.414	0.409	0.025	0.336
	NX	2	0.325	0.00043	0.325	0.156	0.551		3.0	2.042	0.738	0.327	0.416	0.258	0.057
	XJ	2	0.523	0.00069	0.523	1.505	1.405		2.6	1.698	0.580	0.250	0.336	0.317	0.074
	Overall	6	0.401	0.00058	0.441	−0.739	−1.971		3.0	1.988	0.693	0.325	0.382	0.191	0.170
MEAM1	LN	1	—	—	—	—	—		2.6	1.770	0.617	0.320	0.344	0.179	0.627
	ZJ	2	0.125	0.00017	0.125	−1.162	−0.700		3.0	2.117	0.644	0.360	0.330	−0.038	1.000
	FJ	1	—	—	—	—	—		2.6	2.149	0.635	0.343	0.341	0.071	0.420
	GD	1	—	—	—	—	—		3.4	1.951	0.697	0.357	0.350	0.015	0.346
	GX	1	—	—	—	—	—		3.0	1.794	0.585	0.267	0.296	0.141	0.047
	HI	3	0.362	0.0005	0.381	−1.002	−0.918		3.0	2.247	0.736	0.450	0.406	−0.065	0.094
	GS	1	—	—	—	—	—		2.2	1.854	0.480	0.289	0.273	0.000	0.922
	XJ	1	—	—	—	—	—		2.8	1.970	0.681	0.433	0.383	−0.040	0.893
	Overall	4	0.064	0.00009	0.065	−1.529	−5.383		2.8	1.981	0.634	0.352	0.340	0.033	0.543

Abbreviations: *h*, number of haplotypes; *Hd*, haplotype diversity; *π*, nucleotide diversity; *k*, average number of nucleotide differences; *Na*, observed number of alleles; *Ne*: effective number of alleles; *I*, Shannon’s information index; *Ho*, observed heterozygosity; *He*, expected heterozygosity; *F_IS_*, inbreeding coefficient.

**Table 3:** Genetic diversity of five microsatellite loci in the *B. tabaci* MED and MEAM1 populations.

Cryptic Species	Locus	Number of Alleles (*A*)	Number of Homozygotes (*Homs*)	Number of Heterozygotes (*Hets*)	Allele Richness (*A*_R_)	Null Allele Frequency
MED	BEM6	5	249	8	3.517	0.352
	BEM15	7	197	60	2.224	0.003
	BEM18	4	125	132	2.455	0.009
	BEM23	3	230	27	1.605	0.014
	BEM25	9	190	67	4.863	0.015
MEAM1	BEM6	5	71	4	1.742	0.085
	BEM15	7	19	56	3.919	0.000
	BEM18	3	57	18	1.851	0.000
	BEM23	3	72	3	1.259	0.012
	BEM25	7	24	51	4.167	0.027

**Table 4:** Haplotype distribution of *mtCOI* gene sequences in different populations of *B. tabaci* MED in China.

Population	Haplotype Distribution (%)					
	Hap1	Hap2	Hap3	Hap4	Hap5	Hap6
HE	85.71	14.29				
SX	100					
NM	100					
LN	56.00	40.00				4.00
HL	100					
SH	92.86	7.14				
JS	50.00	45.45	4.55			
ZJ	93.33			6.67		
AH	71.43	21.43			7.14	
FJ	88.89	11.11				
JX	90.91			4.55	4.54	
SD	85.72	9.52		4.76		
HA	70.00	30.00				
HB	100					
HN	86.67	13.33				
GX	27.78	72.22				
CQ	7.69	92.31				
SC	100					
YN	14.28	64.29	21.43			
SN	93.33	6.67				
NX	81.25	18.75				
XJ	44.44	55.56				
Total	73.70	23.82	0.99	0.74	0.50	0.25

**Table 5:** Haplotype distribution of *mtCOI* gene sequences in different populations of *B. tabaci* MEAM1 in China.

Population	Haplotype Distribution (%)			
	Hap1	Hap2	Hap3	Hap4
LN	100			
ZJ	93.75	6.25		
FJ	100			
GD	100			
GX	100			
HI	80.00		13.33	6.73
GS	100			
XJ	100			
Total	96.72	0.78	1.67	0.83

**Table 6:** Results of analysis of molecular variance (AMOVA) among different populations of *B. tabaci* MED and MEAM1 in China on the basis of mitochondrial *COI* and microsatellite markers.

Cryptic Species	Marker	Source of Variation	d.f.	Sum of Squares	Variance Components	Percentage of Variation
MED	mitochondrial *COI*	Among populations	21	36.820	0.089	39.5
		Within populations	381	51.740	0.136	60.5
		Total	402	88.561	0.224	
	microsatellites	Among populations	21	86.170	0.133	11.8
		Within populations	492	490.629	0.997	88.2
		Total	513	570.800	1.131	
MEAM1	mitochondrial *COI*	Among populations	7	0.347	0.001	3.7
		Within populations	115	3.604	0.031	96.3
		Total	122	3.951	0.033	
	microsatellites	Among populations	7	11.844	0.043	4.6
		Within populations	142	127.396	0.897	95.4
		Total	149	139.240	0.940	

## Data Availability

All data are contained within the article.
